# T Cell LFA-1 Engagement Induces HuR-Dependent Cytokine mRNA Stabilization through a Vav-1, Rac1/2, p38MAPK and MKK3 Signaling Cascade

**DOI:** 10.1371/journal.pone.0014450

**Published:** 2010-12-29

**Authors:** Vinod S. Ramgolam, Scott D. DeGregorio, Gautham K. Rao, Mark Collinge, Sharmila S. Subaran, Silva Markovic-Plese, Ruggero Pardi, Jeffrey R. Bender

**Affiliations:** 1 Raymond and Beverly Sackler Foundation Cardiovascular Laboratory, Departments of Medicine (Cardiovascular Medicine) and Immunobiology, Vascular Biology and Therapeutics Program, Yale University School of Medicine, New Haven, Connecticut, United States of America; 2 Department of Neurology and of Microbiology and Immunology, University of North Carolina at Chapel Hill, Chapel Hill, North Carolina, United States of America; 3 Department of Molecular Pathology, Universitá Vita-Salute School of Medicine, San Raffaele Scientific Institute, Milan, Italy; New York University, United States of America

## Abstract

**Background:**

Engagement of the β2 integrin, lymphocyte function-associated antigen-1 (LFA-1), results in stabilization of T cell mRNA transcripts containing AU-rich elements (AREs) by inducing rapid nuclear-to-cytosolic translocation of the RNA-stabilizing protein, HuR. However, little is known regarding integrin-induced signaling cascades that affect mRNA catabolism. This study examines the role of the GTPases, Rac 1 and Rac 2, and their downstream effectors, in the LFA-1-induced effects on mRNA.

**Methodology/Principal Findings:**

Engagement of LFA-1 to its ligand, ICAM-1, in human peripheral T cells resulted in rapid activation of Rac1 and Rac2. siRNA-mediated knockdown of either Rac1 or Rac2 prevented LFA-1-stimulated stabilization of the labile transcripts encoding IFN-γ and TNF-α, and integrin mediated IFN-γ mRNA stabilization was absent in T cells obtained from Rac2 gene-deleted mice. LFA-1 engagement-induced translocation of HuR and stabilization of TNF- α mRNA was lost in Jurkat cells deficient in the Rac guanine nucleotide exchange factor Vav-1 (J.Vav1). The transfection of J.Vav1 cells with constitutively active Rac1 or Rac2 stabilized a labile β-globin reporter mRNA, in a HuR-dependent manner. Furthermore, LFA-1-mediated mRNA stabilization and HuR translocation in mouse splenic T cells was dependent on the phosphorylation of the mitogen-activated protein kinase kinase, MKK3, and its target MAP kinase p38MAPK, and lost in T cells obtained from MKK3 gene-deleted mice.

**Conclusions/Significance:**

Collectively, these results demonstrate that LFA-1-induced stabilization of ARE-containing mRNAs in T cells is dependent on HuR, and occurs through the Vav-1, Rac1/2, MKK3 and p38MAPK signaling cascade. This pathway constitutes a molecular switch that enhances immune and pro-inflammatory gene expression in T cells undergoing adhesion at sites of activation and effector function.

## Introduction

Integrins are essential for leukocyte extravasation at sites of inflammation. They direct formation of the immunological synapse and macromolecular complexes consisting of structural as well as signaling proteins. The β2 integrin, lymphocyte function-associated antigen-1 (LFA-1), also known as α_L_β_2_ or CD11a/CD18, is expressed on all cells of hematopoietic lineage, and is important in leukocyte adhesion, locomotion and transendothelial migration. Through “inside-out” signaling, T cell receptor (TCR) engagement induces allosteric transition of LFA-1 to a high-affinity activation state. Upon ligand binding, LFA-1 can then contribute, through “outside-in” signaling, to T cell activation [1]. We have demonstrated that such LFA-1 engagement-triggered transmembrane signaling events can lead to stabilization of otherwise labile mRNA transcripts bearing adenylate- and uridylate-rich elements (AREs) in their 3′-untranslated region (3′-UTR), including those encoding Th1-type cytokines, interferon-γ (IFN-γ) [2] and tumor necrosis factor-α (TNF-α) [3]. The mRNA-stabilizing downstream molecular target of LFA-1 signaling is HuR, an ARE-binding and stabilizing protein that is constitutively expressed in the T cell nucleus. We have recently demonstrated that T cell HuR can undergo rapid nuclear-to-cytoplasmic translocation solely by engagement of LFA-1 with its ligand ICAM-1 [3]. Such altered subcellular localization of HuR has been shown to correlate with its mRNA stabilizing activity [4]. The proximal signaling events involved in LFA-1-stimulated HuR translocation and consequent mRNA stabilization are unknown.

LFA-1-mediated cell adhesion promotes alterations in cell morphology including the generation of lamellipodial extensions required for cell migration [5]. The Rho family of GTP-binding proteins, including Rac, Rho and Cdc42, are key intermediate signal transducers of these dynamic cytoskeletal events in many cell types, including T cells [6]. Rac1 expression is ubiquitous, whereas Rac2 is expressed only in cells of hematopoietic lineage [7]. These Rac1- and Rac2 GTPases share functional roles but also have distinct functions in hematopoietic systems. A study by Guo et al. recently demonstrated that these two GTPases play a redundant role T cell development [8], whereas murine T cells deficient in Rac2 exhibit profound activation defects, with decreased proliferation in response to stimulation with anti-CD3 and anti-CD28 [9]. Furthermore, Rac2 plays a critical role in neutrophil migration, and Rac1 is involved in cell spreading [10].

The activation of Rac1 and Rac2 requires the phosphorylation of guanine nucleotide exchange factors (GEFs), which promote the exchange of GDP for GTP and confer the active state to this group of GTPases. Vav-1 is hematopoietic cell-restricted and serves as a GEF for Rac1, Rac2 and Rho G [11]. LFA-1 engagement-mediated activation of Rac1 occurs through Vav-1 in T lymphoblasts [12]. T cell Rac2 activation also likely occurs through Vav-1-mediated GTP loading.

In addition to Rac's role in cell spreading and motility, Rac-dependent gene expression has been investigated [13], although not as a consequence of β2 integrin engagement. Several transcripts stabilized by LFA-1 engagement encode proteins involved in T cell differentiation and mitogenesis, making the mitogen-activated protein kinases (MAPKs) likely candidates linking integrin engagement, Rac activation and modulation of RNA binding proteins. Extracellular signal regulated protein kinase (ERK), c-jun NH_2_ terminal protein kinase (JNK), and p38MAPK are all abundant in T cells. The ERKs are activated by a Ras-dependent pathway in response to many growth factors and hormones [14]. The JNK and p38MAPK cell signaling pathways are both integral in T cell differentiation [15]. Activation of p38MAPK in naïve T cells causes the release of IL-12, while both p38MAPK and the JNK isoform, JNK2, stimulate differentiation of naïve T cells into Th1 cells, a process involved in IFN-γ synthesis [15], [16]. MKK3, which is directly downstream of Rac1, is the p38-activating MAPK within T cells outside of the thymus [17]. The integration of extracellular signals (LFA-1 engagement) into modulation of the mRNA binding protein, HuR, by any of these MAPKs, has not been studied.

In this study, we demonstrate that Vav-1, Rac1, -2, MKK3 and p38MAPK are proximal and intermediate signaling molecules involved in LFA-1-stimulated mRNA stabilization. LFA-1 engagement induces transient activation of Rac1 and Rac2 in peripheral human T cells. Vav-1 deficiency prevents LFA-1-triggered HuR nuclear-to-cytoplasmic translocation, and consequent labile transcript stabilization. Silencing of either endogenous Rac1 or Rac2 abrogates T cell IFN-γ and TNF-α mRNA stabilization induced upon T cell LFA-1 engagement. Expression of constitutively active, recombinant Rac (Rac1 or Rac2) was sufficient to prolong the half-life of an otherwise labile reporter mRNA in T cells. Furthermore, we demonstrate that LFA-1- induced mRNA stabilization requires the Rac1, 2 downstream effectors MKK3 and p38MAPK. We discuss the relevance of these events in adhesion-dependent T cell activation.

## Results

### LFA-1 engagement induces IFN-γ and TNF-α mRNA binding to HuR

**Figure 1:**
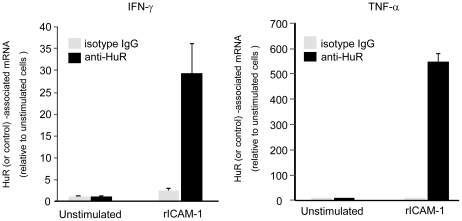
T cell LFA-1-stimulated HuR binding to cytokine mRNAs. Human primary peripheral T cells were unstimulated or PMA-activated (5 ng/ml) on rICAM-1- coated plates for 3 h, after which they were reversibly crosslinked with 1% formaldehyde followed by sonication. HuR (or IgG_1_ control) was immunoprecipitated from sonicated lysates and RNA isolated from immunoprecipitates, followed by reverse transcription and qPCR for IFN-γ, TNF-α and GAPDH to analyze relative mRNA levels. RNA levels detected in unstimulated cell immunoprecipitates was set at 1. RNA levels in immunoprecipitates from ICAM-1-bound cells are relative to those values of 1. One representative of 3 separate experiments is shown. Samples were analyzed in triplicate and data represented as mean ± S.E.M.

We have previously demonstrated that LFA-1 engagement results in HuR nuclear-to-cytoplasmic translocation and cytokine mRNA stabilization [2]. In the current work, we have addressed the signaling pathway responsible for these events. In anticipation of dissecting those molecular signals, we addressed whether LFA-1-induced T cell activation results in direct binding of HuR to IFN-γ and TNF-α mRNA. Freshly isolated, human peripheral T cells were PMA (5 ng/ml) activated and bound to rICAM-1, after which protein-RNA complexes were formaldehyde-crosslinked. Cells were sonicated, HuR immunoprecipitated and RNA isolated from the immunoprecipitates. Figure 1 displays qPCR data from cDNA templates reverse transcribed from IFN-γ and TNF-α mRNA recovered in HuR immunoprecipitates. Controls include the same from unstimulated cells, as well as RNA recovered from isotype-matched irrelevant IgG immunoprecipitates. Relative to that recovered in unstimulated cell HuR immunoprecipitates, levels of HuR-associated IFN-γ and TNF-α mRNA are 29- and 552- fold higher, respectively, in integrin-engaged cells. There is a negligible to minimal transcript increase detected in isotype control IgG immunoprecipates (Fig. 1), as well as in PMA alone-treated cells (not shown). These results demonstrate that T cell LFA-1 engagement results in a dramatic increase in labile cytokine transcript association with HuR.

### LFA-1 engagement induces transient Rac1 and Rac2 activation

**Figure 2:**
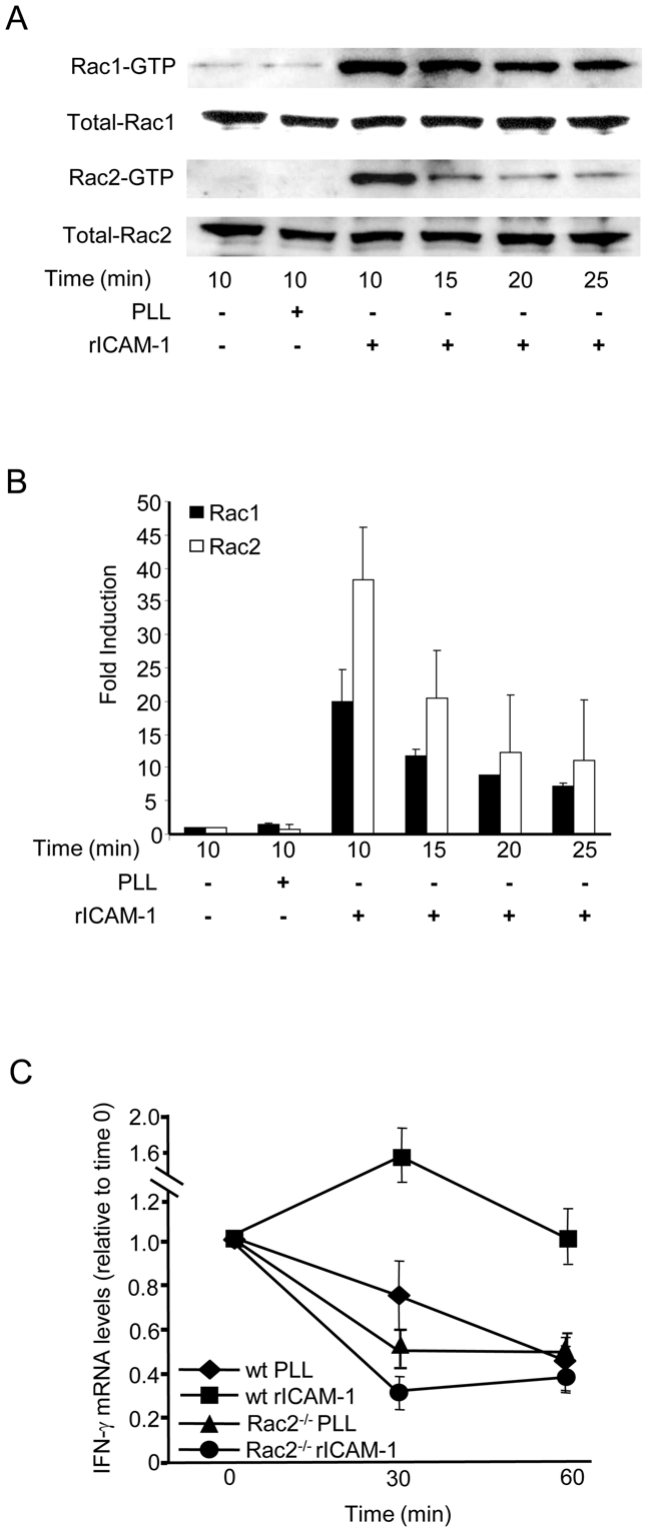
LFA-1 engagement-mediated Rac1 and Rac2 activation in human peripheral T cells. *A*, Peripheral human T cells were treated with MnCl_2_- containing LFA-1 activation buffer and plated onto rICAM-1-coated dishes for the indicated times. Control cells were treated with LFA-1 activation buffer and kept in suspension or plated on PLL-coated dishes for 10 minutes. Cell lysates were recovered at the indicated time points and activated Rac1/2 (Rac1/2-GTP) pulled down using GST-PBD-agarose beads. GST-PBD pull-down samples (upper panels) and total cell extracts (lower panels) were subjected to immunoblotting with anti-Rac1 or anti-Rac2 antibodies. Data are representative of three separate experiments. *B*, Densitometric quantification of immunoblots shown in *A*. Rac1-GTP and Rac2-GTP were normalized to total Rac1 or Rac2 and expressed as relative fold induction compared to Rac1- or Rac2- GTP in LFA-1 activation buffer-treated cells. Histograms represent mean ± standard deviation of three separate experiments. *C*, Mouse splenic T cells were isolated from wt or Rac2^−/−^ mice and were plated on PLL (wt) or rICAM-1- coated dishes, after which they were stimulated with 5 ng/ml PMA for induction of gene transcription and cell adhesion for 3 h. DRB at a concentration of 250 µM was added and mRNA recovered at 0, 30, or 60 min for IFN-γ mRNA decay analysis by qRT-PCR. One representative experiment of 3 seperate experiments is shown. Samples were analyzed in triplicate and data represented as mean ± S.E.M.

T lymphocyte adhesion to β2 integrin ligands results in cell spreading, a consequence of profound changes in cytoskeletal structure and conformation [18]. Our previous T cell co-activation experiments, in which LFA-1 engagement resulted in stabilization of otherwise labile transcripts, included marked spreading when cells bound either to rICAM-1 or through activating anti-LFA-1 antibodies. Small G proteins of the Rho family GTPases, Rac1 and Rac2, are key effectors of these cytoskeletal changes, thereby integrating activation responses generated by LFA-1 transmembrane signaling. To determine whether LFA-1 engagement activates T cell Rac1 and/or Rac2, and whether the kinetics of activation are consistent with rapid modulation of mRNA binding proteins, peripheral human T cells were MnCl_2_-treated, to promote LFA-1 affinity modulation and prime for ICAM-1 binding, after which they were adhered to rICAM-1-coated dishes. Cell extracts were recovered, and activated GTP-bound Rac1 or Rac2 was “pulled down” with a GST-p21-activated kinase (PAK) binding domain (PBD) fusion protein [19]. PBD binds only to activated Rac1 or Rac2, the determination of which is made by immunoblotting with anti-Rac1 or -2. Figure 2A demonstrates that Rac1 and Rac2 activation occurs within 10 minutes, with Rac1- or Rac2-GTP levels waning thereafter. Although both Rac isoforms are rapidly activated, the Rac2 activation was consistently more robust than Rac1 activation (38- vs. 20-fold in Figure 2B). Further evidence of Rac2's (Rac's) critical role in T cell integrin-induced modulation of cytokine mRNA decay comes from our experiments performed with splenic T cells obtained from Rac2 gene-deleted mice. In these experiments, a low concentration of PMA (5 ng/ml) was used to induce IFN-γ transcription and LFA-1 affinity modulation for ICAM-1 binding. Figure 2C demonstrates that the IFN-γ mRNA stabilization imparted by LFA-1 engagement (wt rICAM-1) is absent in Rac2^−/−^ T cells on poly-L-lysine (PLL) and Rac2^−/−^ on rICAM-1. In fact, IFN-γ transcripts in these T cells decay even more rapidly than in control/non-integrin-engaged wild-type T cells (wt vs. Rac2 rICAM-1 at 30 min time point).

### Silencing of Rac1 or -2 abrogates LFA-1-induced mRNA stabilization

**Figure 3:**
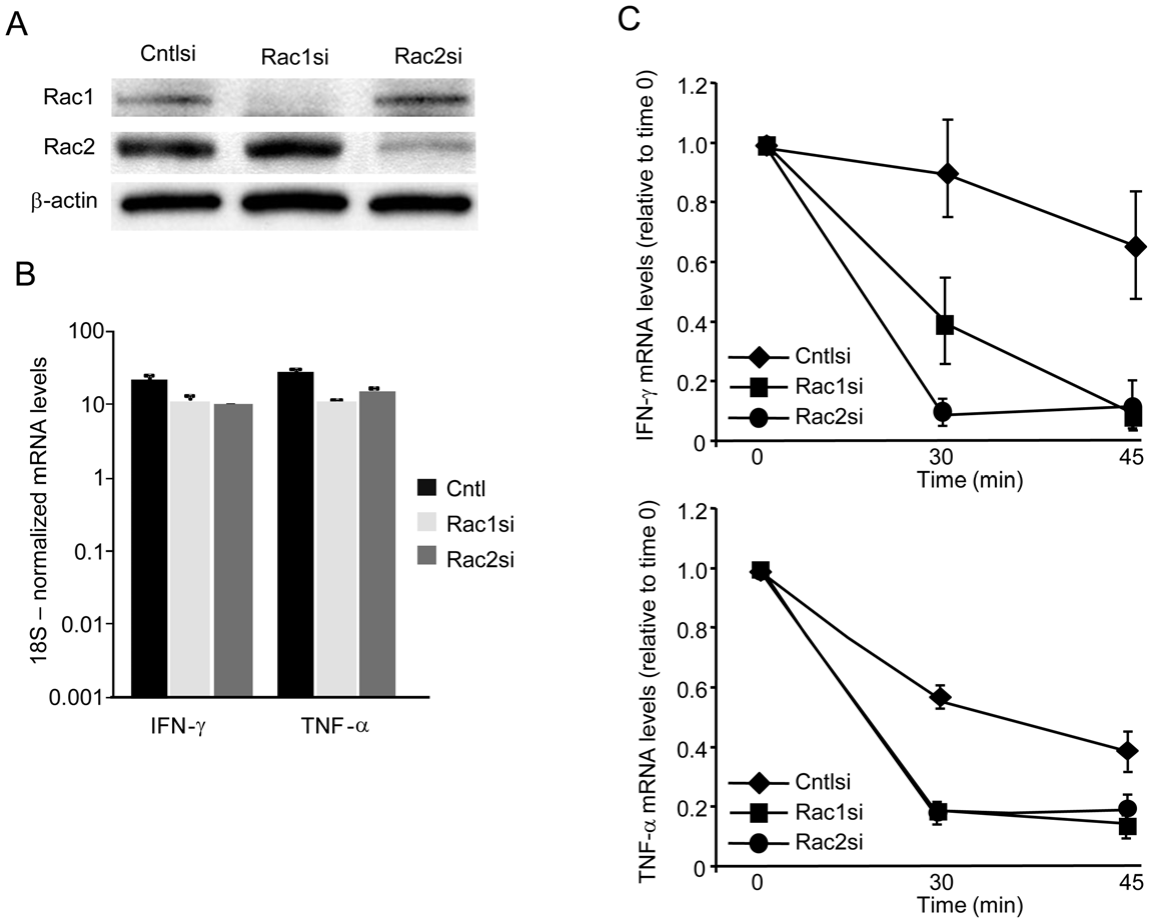
Requirement of Rac1 and Rac2 in LFA-1- induced mRNA stabilization. Human peripheral T cells were transfected with Rac1 (Rac1si), Rac2 (Rac2si) or control scrambled (Cntlsi) siRNA duplex oligonucleotides. *A*, Lysates obtained from siRNA-treated cells were immunoblotted with antibodies to the indicated molecules to determine the knockdown efficacy and specificity. *B*, Human peripheral T cells were treated with the indicated siRNA oligionucleotides, after which they were PMA (5 ng/ml)-activated on rICAM-1-coated plates for 3 h. qPCR for IFN-γ and TNF-α was performed, with mRNA levels shown relative to 18S RNA. *C*, siRNA- treated human peripheral T cells were stimulated with 5 ng/ml PMA and plated on rICAM-1 coated plates for 3 hours, after which 0.2 mM DRB was added and cells were lysed after 0, 30 and 45 mins. IFN-γ and TNF-α mRNA decay was determined by qRT-PCR. Samples were analyzed in triplicate and data presented as the mean ± S.E.M. One representative of 3 independent experiments is shown.

To address which isoform of endogenous Rac is critical in LFA-1-dependent mRNA stabilization, peripheral human T cells were subjected to siRNA-mediated knockdown of Rac1 and Rac2. Figure 3A demonstrates the specific depletion of Rac1 and Rac2. Control, Rac1 or Rac2 knockdown cells were stimulated with PMA, rICAM-1 bound and stability of IFN-γ, and TNF-α mRNA was assessed. PMA-induced increases in transcript levels (*t* = 0) was similar in all groups, confirming that the knockdown cells were viable and transcriptionally active (Figure 3B). LFA-1-induced stabilization of these 2 transcripts was markedly diminished in Rac1 and Rac2 knockdown cells (Fig. 3C). These knockdown results demonstrate that both Rac1 and Rac2 participate in and appear required for LFA-1 engagement-induced stability of the labile IFN-γ and TNF-α mRNA transcripts in the primary T cells.

### Constitutively active Rac1 and 2 induce HuR-dependent mRNA stabilization

**Figure 4:**
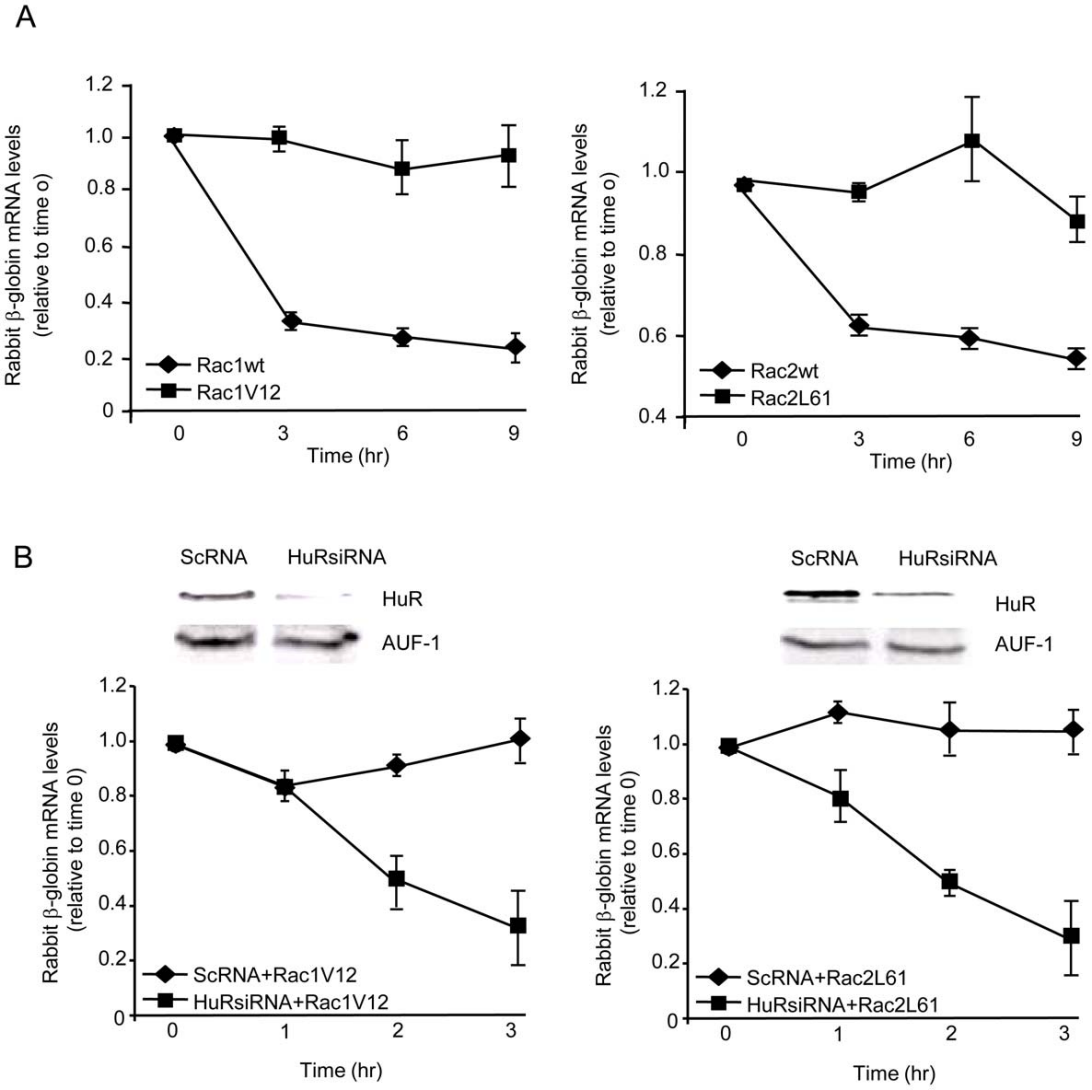
HuR dependence in Rac1- and Rac2- mediated mRNA stabilization. *A*, J.Vav1 cells were co-transfected with the chimeric β-globin/uPAR 3′ARE, CAT, and either wild type (wt) or constitutively active Rac1 (Rac1V12) or Rac2 (Rac2L61) expression constructs. Cells were serum starved for 24 hr, followed by 4 hr serum stimulation (time 0), after which mRNA was harvested at 3, 6, and 9 hr. β-globin mRNA levels, normalized to CAT transcript level, were determined by qRT-PCR. Data are presented as the combined mean ± S.E.M. of three separate experiments. *B*, HuR depletion (knockdown) was performed with an HuR siRNA or ScRNA. Viable cells were recovered on Ficoll gradients and transfected as in *A*, with the Rac1V12 (left panel) or the Rac2L61 (right panel). Immunoblots show effective and specific kockdown of HuR compared with AUF-1, another ARE-binding protein. mRNA was harvested from cells at the indicated time points following 4 hr serum stimulation. β-globin mRNA levels, normalized to CAT transcript level, were determined by qRT-PCR. β-globin mRNA levels are expressed relative to those observed at time 0 (i.e., after 4 hr serum stimulation). Data are presented as the mean ± S.E.M. of three separate experiments.

We have previously demonstrated that LFA-1 drives the stabilization of mRNA transcripts encoding either class I or class II ARE sequences [2]. Since our findings above indicated that LFA-1 engagement results in Rac1 and Rac2 activation and that Rac1 and Rac2 are required for mRNA stabilization, we investigated whether Rac activation is sufficient to promote mRNA stabilization. We employed a class II encoding reporter construct to study the effect of Rac activation on mRNA stability. Vav-1 deficient Jurkat (J.Vav1) cells were transfected with constitutively active Rac1 (Rac1 V12) or Rac2 (Rac2 L61) [20], and their effect on a co-transfected mRNA reporter were evaluated. The mRNA reporter construct, pBBB-3′-uPAR, encodes rabbit β-globin mRNA, with the uPAR 3′-UTR ARE cloned into the 3′ position, conferring instability to this otherwise stable transcript [2]. Cells were also co-transfected with a CAT expression construct, allowing for normalization. The rabbit β-globin cDNA, under the control of the c-fos promoter, was serum-induced in transfected cells for 4 hr, after which mRNA was harvested at 3, 6 and 9 hr. The ARE-containing β-globin mRNA decays progressively over a 9 hr period, with the most significant degradation occurring in the first 3 hr. Figure 4A demonstrates that over-expression of wild-type Rac1 or Rac2 does not prevent degradation of the reporter mRNA. However, expression of constitutively active Rac1 (V12) or Rac2 (L61) is sufficient to confer stability to the β-globin transcript. These data indicate that, when expressed in a human T cell line, active Rac1 or Rac2 is sufficient to drive stabilization of an ARE-containing mRNA.

To document the link between T cell HuR and Rac-mediated mRNA stabilization, siRNA-mediated knockdown was performed using duplex oligonucleotides we have previously shown to effectively reduce HuR levels in T cells. Figure 4B demonstrates a siRNA-mediated reduction of HuR levels of 90% and 80% in J.Vav1, when compared with control (ScRNA) oligonucleotides, and no effect on levels of another ARE-binding protein, AUF-1. The β-globin-ARE stabilizing effect of constitutively active Rac1 and Rac2 was lost in cells with the noted marked reduction of HuR levels (Fig. 4B). These findings demonstrate that active Rac-mediated stabilization of ARE-bearing transcripts is HuR-dependent in T cells.

### LFA-1-stimulated HuR translocation and mRNA stabilization is Vav-1-dependent

**Figure 5:**
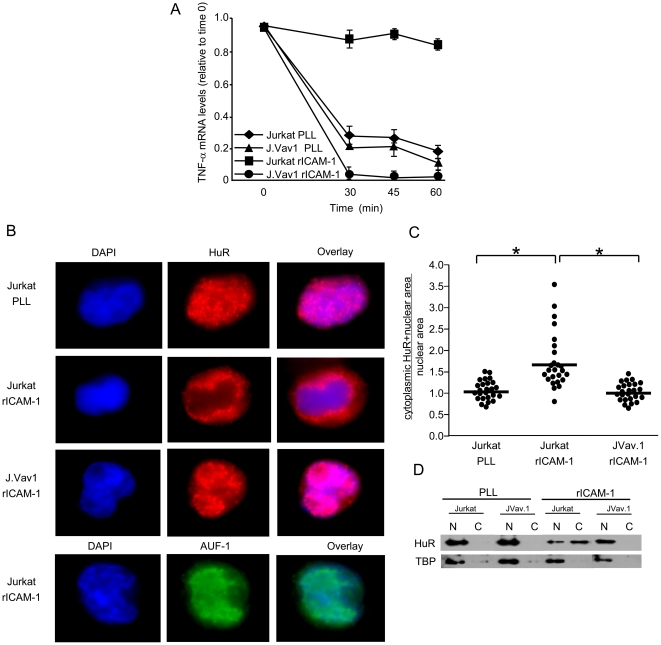
Role of Vav-1 in LFA-1-mediated TNF-α mRNA stabilization and HuR translocation. *A*, Jurkat or J.Vav1 cells were plated onto rICAM-1- or PLL coated dishes for 4 hours in the presence of 5 ng/ml PMA. Cells were transcription-arrested with DRB (0.2 mM, time 0) and RNA harvested at the indicated time points for TNF-α qRT-PCR analysis. TNF-α mRNA levels were normalized to GAPDH and are expressed relative to time 0. Data are presented as the mean ± SEM of three separate experiments. *B*, Jurkat or J.Vav1 cells were induced for LFA-1 activation with MnCl_2_ and adhered to rICAM-1- or control PLL coated coverslips at 37°C for 60 min. Cells were fixed, permeabilized, and immunofluorescent costaining was performed with mouse anti-human HuR or AUF-1 and rabbit anti-mouse Ig-cyanine 5, and with DAPI for nuclear definition. Individual images were overlaid and merges displayed as noted. Magnification 100×. *C*, 25 cells in 3 different fields were morphometrically analyzed for HuR nuclear-to-cytosolic translocation and expressed as the ratio of HuR immunofluorescent plus nuclear area divided by the nuclear area only, analyzed by Image J. Each dot represents a single cell ratio. *p<0.05. *D*, Jurkat cells and JVav.1 cells were treated as described above, after which nuclear (N) and cytosolic (C) fractions were isolated, followed by immunoblotting for HuR and TBP. A representative blot of 3 separate experiments is shown.

The GEF, Vav-1, is a Rac activator in hematopoietic cells, and is abundantly expressed in T cells [21], [22]. Because Vav-1 activation in T cells has been demonstrated upon LFA-1 engagement [12], we further used the J.Vav1 cells to address the role of Vav-1 in LFA-1-mediated mRNA stabilization. The TNF-α mRNA stabilization observed in wt Jurkat cells adhered to rICAM-1 was absent in J.Vav1 cells (Fig. 5A, Jurkat PMA/rICAM-1 vs. J.Vav1 PMA/rICAM-1), with minimal detectable TNF-α mRNA at 60 min in the LFA-1-stimulated J.Vav1 cells, similar to wild-type Jurkat activated with PMA alone.

These results demonstrate that Vav-1 (likely GEF activity) is required for T cell LFA-1-induced stabilization of cytokine transcripts bearing AREs, such as those encoding TNF-α and IFN-γ. We have previously shown that β2 integrin-induced TNF-αmRNA stabilization leads to augmented production of TNF-α protein [3]. We have also demonstrated that T cell LFA-1 engagement leads to nuclear-to-cytoplasmic translocation of the ARE-binding and mRNA-stabilizing protein HuR, and that HuR is required for integrin-induced stabilization to occur [3]. To confirm that those molecular events required for LFA-1-induced mRNA stabilization were also required for HuR translocation, immunofluorescent HuR localization analysis was performed on wild-type Jurkat or J.Vav1 cells adhered to immobilized rICAM-1. Figure 5B displays a robust nuclear-to-cytoplasmic HuR translocation within 60 min of MnCl_2_-treated Jurkat binding to rICAM-1, compared to PLL control. Not all predominantly nuclear proteins translocate upon T cell integrin engagement, as AUF-1, another mRNA-binding protein, fails to do so. MnCl_2_-treated J.Vav1 cells, although adhering to rICAM-1, were unable to generate signals resulting in HuR translocation. In these HuR translocation experiments, there is always a range of degree to which HuR exits the nucleus. The example demonstrated by immunofluorescence microscopy in figure 5B is the maximal degree of activation-induced translocation, in which all detectable HuR becomes cytosolic. Because of this range, from minimal to complete, we developed a quantification assay that compares the extent of HuR distribution to the nuclear definition, morphometrically detected and Image J-analyzed. Figure 5C displays data from 25 cells in each experimental group, demonstrating statistically significant HuR translocation in Jurkat, but not JVav.1, cells bound to rICAM-1 compared to PLL. This is further confirmed biochemically, by the detection of HuR in cytosolic extracts obtained from Jurkat, but not JVav.1, cells bound to rICAM-1 (Fig. 5D). These findings demonstrate that the guanine nucleotide exchange factor Vav-1 is required for T cell integrin-stimuated HuR translocation and labile transcript stabilization.

### LFA-1 mediated mRNA stabilization and HuR translocation is MKK3 dependent

**Figure 6:**
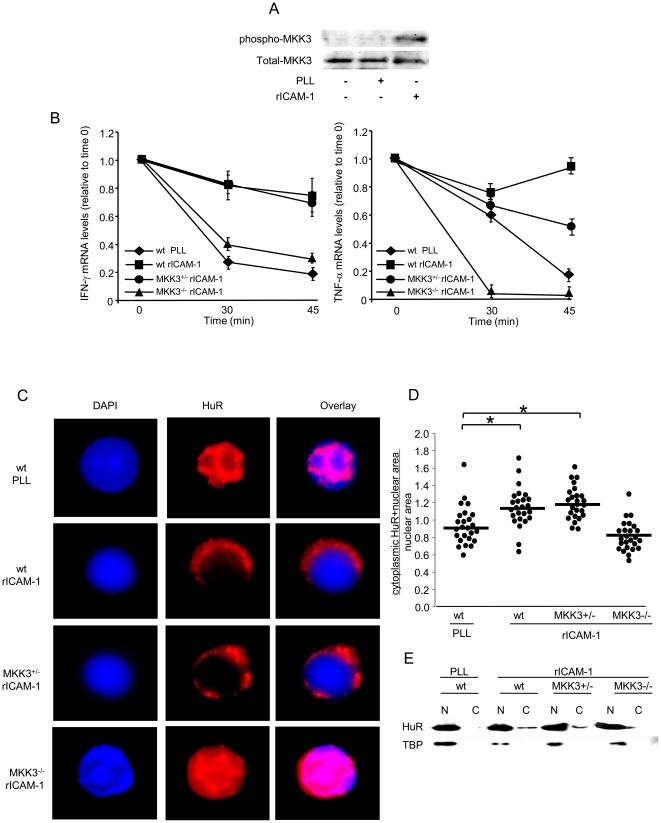
Effect of MKK3 gene deletion on LFA-1 engagement-mediated mRNA stabilization and HuR translocation. *A*, LFA-1 activation buffer-treated human peripheral T cells were adhered onto PLL - or rICAM-1 coated dishes for 30 mins, after which lysates were immunoblotted for activation-specific phospho-MKK3 and total MKK3 (representative of 3 separate experiments). *B*, Murine splenic T cells purified from wt, MKK3^+/−^, and MKK3^−/−^ mice were stimulated with 5 ng/ml PMA and plated on rICAM-1-coated dishes or PLL control (wt). Transcription was arrested with DRB 0.2 mM after 3 hours and RNA harvested at the indicated time points. IFN-γ and TNF-α mRNA levels were determined by qRT-PCR and expressed as fold induction relative to time 0. Data are presented as the mean ± SEM. *C*, T cells were treated with LFA-1 activation buffer and adhered to PLL- or rICAM-1-coated coverslips for 60 minutes. Immunofluorescent analysis was performed with anti-HuR mAb (red) and the nuclear marker DAPI (blue). Magnification 100X. *D*, 25 cells in 3 different fields were morphometrically analyzed for HuR nuclear-to-cytosolic translocation and expressed as the ratio of HuR immunofluorescent plus nuclear area divided by the nuclear area only, analyzed by Image J. Each dot represents a single cell ratio. *p<0.05. *E*, Nuclear (N) and cytosolic (C) fractions were isolated, followed by immunoblotting for HuR and TBP. One representative of 3 seperate experiments is shown.

Rac knockdown reduces LFA-1-induced phosphorylation of the Rac effector MKK3 (data not shown), which is otherwise marked when human peripheral blood T cells adhere to ICAM-1(Fig. 6A). We used a genetic approach to determine whether LFA-1, Vav/Rac-induced cytokine mRNA stabilization requires MKK3. Splenic T cells derived from wt, MKK3^+/−^, or MKK3^−/−^ mice were stimulated with PMA on PLL or rICAM-1 coated plates, followed by transcription arrest and IFN-γ and TNF-α mRNA decay analysis. MKK3 was undetectable in the MKK3^−/−^ cells, whereas its level was reduced (∼50%) in cells obtained from the heterozygote mice (not shown). As expected, LFA-1 engagement (adhesion to rICAM-1) markedly extends the half-life of IFN-γ and TNF-α mRNA in MKK3^+/+^ (wt) T cells (Fig. 6B). This integrin-induced cytokine mRNA stabilization is lost in T cells obtained from MKK3 gene-deleted (MKK3^−/−^) mice, with 45 min transcript decay levels nearly identical to those recovered from MKK3^+/+^ mouse T cells adhered to PLL. The reduced MKK3 levels in MKK3^+/−^ T cells are sufficient to maintain integrin-stimulated signaling resulting in RNA stability, as 45 min cytokine transcript levels recovered from MKK3^+/−^ T cells adhered to rICAM-1 are either identical to (IFN-γ) or only slightly reduced from (TNF-α) wt T cell levels (Fig. 6B). HuR translocation asays followed the same pattern. Figure 6C demonstrates loss of LFA-1-induced HuR nuclear-to-cytosolic translocation in MKK3^−/−^ T cells, whereas this apparent HuR modulation and change in subcellular compartment is maintained in MKK3^−/−^ T cells. This is quantitatively displayed in figure 6D, and confirmed biochemically by immunoblot detection of cytosolic HuR in figure 6E. All these results demonstrate that the Rac-activated MKK3 is required for LFA-1-induced T cell HuR translocation and consequent labile cytokine mRNA stabilization.

### p38MAPK is a key effector in LFA-1-induced, Rac2-dependent mRNA stabilization

**Figure 7:**
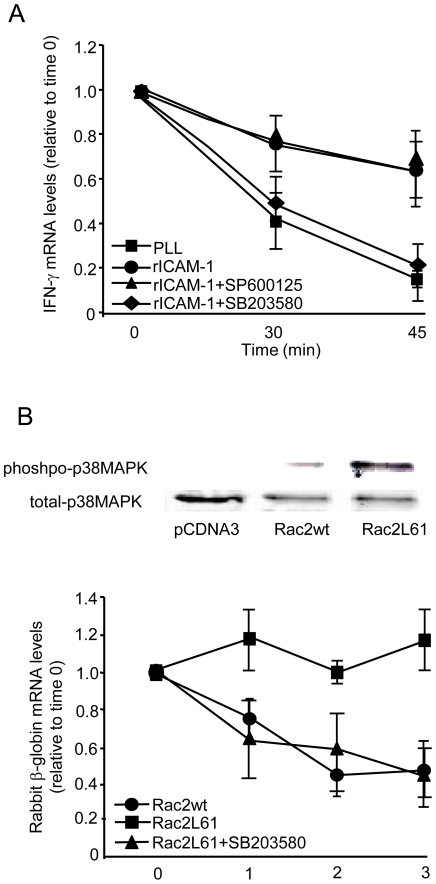
p38MAPK activation requirement in LFA-1- and Rac2- induced mRNA Stabilization. *A*, Murine splenic T cells were pretreated with 10 µM SP600125, 1 µM SB203850 or vehicle control, stimulated with PMA 5 ng/ml and plated on PLL - or rICAM-1- coated dishes. Cells were transcription-arrested with DRB 0.2 mM (time 0) and RNA was harvested at 30 and 45 minutes. IFN-γ mRNA decay was determined by qRT-PCR and expressed as fold induction relative to time 0, normalized to GAPDH mRNA levels. *B*, In addition to the labile chimeric β-globin/uPAR 3′ ARE reporter and CAT constructs, J.Vav1 cells were transfected with pCDNA3, Rac2wt or Rac2L61 constructs. Prior to serum-driven transcriptional activation, cells were pretreated with µM SB203850 or vehicle control for 1 h. mRNA was harvested at the indicated time points (post-serum induction) and β-globin transcript levels were determined by qRT-PCR, shown relative to time 0 and CAT mRNA-normalized. Also shown is activation-specific phospho-p38 (and total p38) immunoblot in the various transfected samples. Data are presented as the mean ± S.E.M. of three separate experiments.

LFA-1 engagement activates both the p38 MAPK and JNK pathways (data not shown), and both can be activated by the upstream kinase MKK3. It is possible that p38, JNK, both, or neither mediate integrin-induced mRNA stabilization. To analyze whether p38 or JNK are necessary for LFA-1-mediated mRNA stabilization, we used the p38 and JNK inhibitors, SB203580 [23] and SP600125 [24], respectively. Both competitively block the ATP binding site of the respective kinase. Murine splenic T cells were stimulated with PMA on PLL plates, or plated on rICAM-1 in the presence or absence of SB203580 or SP600125. SP600125 had no effect on the LFA-1- mediated IFN-γ mRNA stabilization 60 minutes after transcriptional arrest, whereas IFN-γ transcripts decayed in SB2038580 pretreated cells, similar to the decay observed over 60 minutes of T cell binding to PLL control (Fig. 7A). PMA-induced transcriptional activation (i.e., augmented mRNA levels) was intact in the SB2038580 and SP600125 pretreated cells, confirming that the SB2038580 effect was not a toxic one, paralyzing the transcriptional machinery (not shown). These data support a role for p38, but not JNK, in T cell LFA-1-induced cytokine mRNA stabilization.

To complete the link between LFA-1-induced Rac activation, p38 and ARE-bearing stabilization, labile reporter RNA experiments were performed in SB203580-pretreated J.Vav1 cells transfected with either wild-type (non-active) or constitutively active Rac2 (Rac2L61) constructs. Figure 7B displays an active Rac2- induced p38 phosphorylation. These data demonstrate that p38 activation is required for labile transcript stabilization induced by active Rac2.

## Discussion

We have previously demonstrated that LFA-1 engagement, as a co-activation stimulus in human T cells, promotes stabilization of class I and class II ARE-bearing transcripts encoding activation molecules and cytokines [2], [3]. We have described a major role for the ARE-binding and mRNA-stabilizing protein, HuR, in this phenomenon [3]. This dynamically regulated mRNA stabilization event represents a general mechanism whereby adhesion receptor, accessory, co-activator or co-stimulator transmembrane signaling enhances T cell activation responses. With regard to LFA-1, this would represent a novel consequence of integrin “outside-in” signaling. Although the elaboration of second messengers and activation of numerous cascades, following LFA-1 engagement, have been described [5], [18], [25]–[27], this study is focused on those LFA-1-triggered events that direct labile mRNA stabilization.

Members of the Rho GTPase family are known to integrate a variety of signals that occur when extracellular stimuli induce integrin-mediated transmembrane activation responses [28]. Striking features of T cell:APC conjugate formation, or activated T cell arrest on post-capillary venular endothelium, are cell spreading and lamellipodia formation, with consequences of an organized immune synapse or transendothelial migration. Although these events, all involving the actin-based cytoskeleton with microfilament assembly, are thought to be the primary and necessary consequences of LFA-1 engagement in T cells, the array of induced signaling responses and numerous downstream effectors make it likely that these small G proteins are directly involved in other integrin-stimulated cellular events. In our pulldown screening assays performed with primary human T cells, we did not observe an LFA-1-induced Cdc42 activation. Furthermore, the c3 exoenzyme RhoA inhibitor had no effect on LFA-1-unduced cytokine mRNA stabilization. Therefore, we directed our attention to Rac1, Rac2 and their GEF, Vav-1, comparing LFA-1-induced Rac2 and Rac1 activation responses, and the role of each in labile transcript stabilization. Both are rapidly activated by LFA-1 engagement in T cells, although we consistently observed a much greater degree of Rac2 than Rac1 activation in fresh peripheral human T cells. The potential role of either or both is supported by the heterologous experiments using an RNA reporter system, in which expression of either constitutively active Rac2 or Rac1 was sufficient to stabilize the otherwise destabilized, uPAR class II ARE-bearing β-globin transcript. At the least, these experiments demonstrate that both GTPases can act independently to induce mRNA stabilization, when overexpressed. By contrast, siRNA-mediated knockdown of *either* Rac1 or Rac2 abrogated LFA-1-induced mRNA stability in human peripheral T cells suggesting that both Rac1 and Rac2 are necessary. It is possible that in cells which endogenously express low levels of both Rac1 and -2, the threshold level of Rac activation downstream of LFA-1 engagement is not reached upon knockdown of either Rac isoform. Rac1 and Rac2 share 92% sequence identity and many (perhaps identical) targets, including multiple members of the MAP kinase family and their upstream kinases [28]. However, Rac1 and Rac2 are not completely redundant, as they have distinct functions in the innate immune system [28], [29].Together, our current findings with heterologous expression and siRNA-mediated knockdown of Rac1 and -2 show that both Racs are involved and have overlapping function in HuR modulation and mRNA stabilization, with their involvement in the signaling cascade dependent on the level of expression and activation in a given cell.

We believe that engagement of other adhesion receptors can provide similar effects on gene expression. We have previously described that engagement of the T cell β1 integrin, VLA-4, leads to enhancement of membrane uPAR expression, nearly identical to that seen with LFA-1 engagement [30]. uPAR is encoded by a labile mRNA, the half-life of which is prolonged by β2 integrin engagement [2]. In fact, Vav-1 tyrosine phosphorylation and activation have been demonstrated following fibronectin-mediated β1 integrin engagement in Jurkat cells [31]. Although we have not addressed effects on Vav-1-dependent Rac activation, we have previously described that CD28 engagement has similar effects on HuR localization and RNA binding [2]. We now demonstrate Vav-1-dependent effect(s) of LFA-1 engagement on the TNF-α mRNA half-life. Thus, we believe that, upon TCR engagement and transcriptional activation, required “second signals” include those that prolong half-lives of many critical activation transcripts, and that this can be achieved through transmembrane signaling by a variety of molecules, including integrins.

Previous studies have demonstrated a link between the p38 MAPK pathway and cytokine mRNA stabilization [32]. Furthermore, MKK3 and the p38-activated kinase MAPKAPK2 (MK2) were shown to be involved in ARE-mediated mRNA stabilization in a Rac dependent manner in breast cancer cells [33]. HuR was implicated as a mediator of MK2-induced uPAR mRNA stabilization, suggesting a link between the p38 activation pathway, HuR translocation and inflammatory transcript stabilization [4]. p38- and HuR-dependent stabilization of labile transcripts encoding cell cycle regulators has recently been described [34]. Our work is the first to demonstrate a mechanistic link between the engagement of a key adhesion receptor in T lymphocytes, LFA-1, and the -activated MKK3/p38 pathway leading to HuR modulation and consequent immune/inflammatory transcript stabilization. Furthermore, we demonstrate these activation events not only in transformed T cell lines, but also in primary T cells freshly isolated from human peripheral blood and mouse spleen.

In our RNA decay assay, transcript levels sufficient to interpret RNA stability are required at the time of transcriptional arrest. In previous work, we have activated the TCR/CD3 complex with anti-CD3 antibody [2], [3], which does trigger IFN-γ and TNF-α gene transcription. To simplify our protocol, we used a low concentration of PMA in this work. Because PMA-induced HuR translocation and RNA stabilization has been described [35]–[37], it was important to determine whether phorbol ester treatment confounded our analysis of integrin-stimulated effects on T cell HuR and mRNA half-life. We used a concentration range (5–25 ng/ml) in this work that is significantly lower than that used in previous reports. More importantly, a “PMA alone” control was used in all our decay assays and in those experimental samples, the cytokine mRNAs always rapidly decayed (Figs. 2, 3, 5, 6, 7). Finally, we confirmed that this PMA concentration does not induce T cell HuR translocation (Figure S1). Therefore, we are confident that the HuR modulation and consequent RNA effects are integrin-induced.

The presence of destabilizing AREs within IFN-γ and TNF-α mRNA has previously been described. We now demonstrate LFA-1-induced binding of HuR to these labile transcripts. Since leukocyte recruitment, adhesion and migration into tissues are key pathogenetic components of many immune and inflammatory disease states, it is very likely that integrin-mediated mRNA stabilization of these cytokine transcripts enhances pathologically relevant gene expression. Rac-induced immune and inflammatory transcript stabilization could be a therapeutic target in the aforementioned diseases. HMG-CoA reductase inhibitors (statins) block the rate-limiting enzyme in the cholesterol synthesis pathway, preventing mevalonate production. They are used extensively as cholesterol lowering agents. In addition to cholesterol, mevalonate is the precursor to several other important lipid intermediates, including farnesyl pyrophosphate, from which isoprenoids are derived. This includes geranylgeranlyl pyrophosphate, a lipid modification of Rac1 and Rac2 required for their subcellular targeting and function. We have recently found that statin pretreatment inhibits LFA-1-induced Rac2 activation (D. Smith, V. Ramgolam, J. Bender, unpublished). We are currently investigating whether an important statin effect occurs at the level of adhesion-induced, Rac-mediated mRNA stabilization.

In summary, our data support a model for LFA-1-regulated, Vav-1, Rac1 and Rac2, MKK3 and p38MAPK dependent HuR modulation, resulting in marked stabilization of ARE-bearing transcripts, including those encoding IFN-γ and TNF-α. Dissecting the critical Rac effectors and posttranslational alterations on mRNA binding proteins (most notably HuR) will provide important clues to the molecular basis of T cell differentiation and effector responses.

## Materials and Methods

### Reagents

Anti-phospho MKK3, anti-MKK3, Anti-p38MAPK, and phospho-p38MAPK Abs were purchased from Cell Signaling (Beverly, MA), anti-Rac2 Ab was purchased from Abcam (Cambridge, MA), anti-Rac1 Ab was obtained from Upstate (Upstate, NY) anti-AUF-1 and IgG1 isotype control was purchased from Santa Cruz (Santa Cruz, CA). Anti-HuR (clone 3A2) mouse mAb was a kind gift from Dr. Joan Steitz (Yale University, New Haven, CT). Goat anti-human IgG, Fcγ fragment specific, was purchased from Jackson ImmunoResearch (West Grove, PA) and rICAM-1/Fcγ fragment chimeric protein from R&D Systems (Minneapolis, MN). The GST-p21-binding domain (GST-PBD) fusion protein was purchased from Millipore (Temecula, CA), Phorbol myristate acetate (PMA), Poly-L-lysine, 5,6-dichloro-1-β-D-ribobenzimidazole (DRB), Ficoll-Histopaque 1077 and DAPI were all purchased from Sigma (St. Louis, MO). The RNase inhibitor rRNasin was purchased from Promega (Madison, WI). The pharmacological inhibitors SB203580 and SP600125 were purchased from Calbiochem (San Diego, CA). The murine soluble recombinant ICAM-1 producing cell line, NS-1, was a generous gift from Dr. F. Takei (University of British Columbia, Vancouver, Canada) and murine recombinant ICAM-1 was purified as described [38]. The Rac2 wt and constitutively active Rac2 constructs were gifts from Dr. Hongbo Chi (Yale University, New Haven, CT). The pBBB-3′uPAR and pGEF-BOS-CAT plasmids have been described in our previous work [2]. The siRNA duplex used to specifically target HuR, and the control scrambled siRNA duplex, have previously been described [3]. siRNA duplexes used to target Rac1 and Rac2 were custom synthesized from Qiagen (Valencia, CA), and their sequences are as follows: Rac1 siRNA: GUGAAGAAGAGGAAGAGAA; Rac2 siRNA: CCCAACUCAACCUGCUUAA. For the detection of human IFN-γ and TNF-α and 18S in the Rac1 and Rac2 silencing experiments, Taqman probes were purchased from Applied Biosystems and analyzed on a ABI-PRISM 7700 Sequence System (Applied Biosystems).The following qRT- PCR primers were synthesized by the Keck Biotechnology Resource Facility at Yale University, and their sequences are as follows: CAT sense: 5′-GGATAGTTTCACCCTTGTT-3′; CAT antisense: 5′-GATTGGCTGAGACGAAAAAC-3′; Rabbit β-globin sense: 5′-TGGTTGTCTACCCATGGACC-3′; Rabbit β-globin antisense: 5′-ATCCACGTGCAGCTTGTCAC-3′; human TNF-α sense 5′-GTCAGATCATCTTCTCGAAC-3′; human TNF-α antisense: 5′-TGAGGGTTTGCTACAA-3′; mouse GAPDH sense: 5′-CAACTACATGGTTTACATGT-3′; mouse GAPDH antisense: 5′-GGGATCTCGCTCCTGGAAGA-3′; mouse IFN-γ sense: 5′-AGAAATATTTTAATGCAGGT-3′; mouse IFN-γ antisense: 5′-AGTCAGTTACCGAATAATTA-3′; mouse TNF-α sense 5′-CACGTCGTAGCAAACCACCAA-3′; mouse TNF-α antisense 5′-AGCAAATCGGCTGACGGTGT-3′

### Isolation of Human Peripheral T cells

Peripheral blood samples were collected upon obtaining institutional review board-approved (University of North Carolina at Chapel Hill Institutional Review Board (IRB) written informed consent from donors. Human peripheral blood mononuclear cells were isolated from normal volunteers by Ficoll-Histopaque 1077 density centrifugation. T cells were purified from PBMCs using a negative isolation kit from Miltenyi (Auburn, CA). We obtained a consistent purity of >95% as determined by Flow cytometry analysis for CD3 positive cells.

### Rac-GTP Affinity (pulldown) Assay

Rac1 and Rac2 activity was measured by the affinity-precipitation assay based on the specific interaction of the activated Rho-GTPases with their downstream effectors [19]. The Rac-binding domain from p21-activated kinase was obtained as a GST fusion protein (GST-PBD). Following stimulation for different times, non-adhered cells were aspirated and adherent cells were lysed in 500 µl of lysis buffer (50 mM Tris-HCl, pH 7.0, 150 mM NaCl, 5 mM MgCl_2_, 5% glycerol, 0.5% NP-40) supplemented with a protease inhibitor cocktail (Roche Molecular Biochemicals, Germany). 40 µl of each lysate was set aside for total Rac1/2 analysis. To the remainder of the sample was added 10 µg of GST-PBD beads, and following 60 min incubation at 4°C the GST-PBD-Rac-GTP bound beads were washed 3 times with lysis buffer supplemented with protease inhibitor cocktail. Rac1/2 in total cell extracts and in GST-PBD precipitated samples was detected by Western blot analysis and immunoblotted with either anti-Rac1 or anti-Rac2 antibodies. Membranes were stripped after each Ab treatment with Restore stripping buffer (Thermo Scientific).

### Cell Culture and Transient Transfection

Human peripheral T cells were transfected with siRNA oligos by electroporation (AMAXA, Walkersville, MD). T cells were transfected with 100 nM Rac1 siRNA, 50 nM Rac2 siRNA, and 100 nM scrambled sequence of the Rac2 oligos, which served as a control. The depletion of the respective Rac isoforms was determined by Western blot analysis after 48 h of electroporation. Cells were stimulated with 5 ng/ml PMA and plated on rICAM-1 coated dishes for mRNA stabilization experiments.

Jurkat T cells were obtained from the American Type Culture Collection. The Vav-1 deficient Jurkat cell line, J.Vav1, was a gift from Dr. Abraham (The Burnham Institute, San Diego, CA). Jurkat, and J.Vav1, cells were cultured in RPMI-1640 medium (Gibco, Grand Island, NY) supplemented with 10% fetal bovine serum, 2 mM glutamine, and 100 U/ml each of penicillin G and streptomycin. For transient transfection experiments, 2×10^7^ J.Vav1 cells were combined with 75 µg of DNA (25 µg each of three plasmids) and electroporated at 960 µF/250V (Gene Pulser, Bio-Rad, CA). Cells were cultured overnight in 10 ml of media containing 0.5% FBS and 2 mM L-glutamine.

### T Cell Adhesion and cytokine mRNA Decay Assay

Plates were coated with human rICAM-1 as follows: petri dishes or glass cover slips were coated with 10 µg/ml goat anti-human IgG, Fcγ fragment specific, in 50 mM Tris-HCl, pH 9.5 for 1 hr, and subsequently blocked for 1 hr with PBS containing 2% dialyzed FBS followed by incubation overnight at 4°C with 100 ng/ml of rICAM-1-Fcγ fragment chimeric protein. For mouse T cell experiments, dishes or glass coverslips were treated with 2.5 µg/ml murine rICAM-1 in PBS overnight at 4°C. Cells were resuspended to 10^7^/ml in 100 mM Tris-HCl, pH 7.5, 0.9% NaCl, 2 mM MnCl_2_, 2 mM MgCl_2_, 5 mM D-glucose, 1.5% BSA, for LFA-1 activation (affinity modulation), and plated on immobilized rICAM-1 for the indicated times and treatments. Control dishes or glass cover slips were coated with 20 µg/ml PLL overnight at 4°C after which cells were pre-adhered for 30 min at 4°C. To trigger transcription prior to mRNA decay analysis, cells were stimulated with 25 ng/ml (Jurkat and J.Vav1), 5 ng/ml (human peripheral T cells) and 5 ng/ml (murine splenic T cells) PMA for 3 hr on rICAM-1 or PLL coated dishes. Transcription was then inhibited (t = 0) by addition of 2 mM DRB after which RNA was harvested at 15 or 20 min intervals for up to 60 min. mRNA expression was analyzed by qRT-PCR as previously described [3].The expression of IFN-γ and TNF-α in human peripheral T cells was normalized against 18S. The expression of TNF-α or IFN-γ transcript levels in Jurkat, J.Vav1 and murine splenic T cells was normalized to GAPDH mRNA.

### β-globin mRNA Decay Assay

Cells were co-transfected by electroporation with serum-inducible plasmids encoding rabbit β-globin containing the ARE within the 3′UTR of urokinase plasminogen activator receptor (uPAR) (pBBB-3′ uPAR) [2] along with pGEF-BOS-CAT, and either Rac1 or Rac2 wild-type, or constitutively active, constructs. Cells were serum depleted for 24 hr, then repleted with 10% FBS for 4 hr at which time pBBB-3′uPAR expression is maximal (t = 0). RNA was harvested from cells at various time points following 4 hr exposure to serum. β-globin mRNA expression was normalized to CAT mRNA expression by quantitative real-time PCR, as previously described [2].

### Immunoprecipitation of Protein-RNA complexes

Primary human T cells (10^7^ per experimental condition) were stimulated with 5 ng/ml PMA on rICAM-1 coated wells. T cells were collected, washed, and resuspended in CMF-PBS. Cells were fixed for 10 min in 1% formaldehyde, lysed by sonication at 70% with a Sonic Dismembrator 500 (Fisher Scientific, Pittsburgh, PA) in immunoprecipation buffer (25 mM HEPES pH 8.0, 150 mM KCL, 2.5 mM EDTA, 840 µg/ml NaF, 1 mM DTT, 0.1% NP 40) containing EDTA-free complete protease inhibitors and 2 U/µl RNAsin. The lysates were immunoprecipitated with either isotype control (IgG1) or HuR antibodies pre-conjugated to protein A/G-plus agarose beads overnight at 4°C, after which immunoprecipates were treated with 30 µg/ml of proteinase K for 60 mins at 55°C. RNA was isolated using Qiagen RNeasy Mini kit and reverse transcribed. Samples were analyzed in triplicate for expression of IFN-γ and TNF-α mRNA levels, normalized to GAPDH.

### Subellular fractionation

Cells were resuspended in buffer A (10 mM Tris [pH 7.4], 5 mM MgCl_2_, 1.5 mM KOAc, and 2 mM DTT), rocked at 4°C for 10 min followed by centrifugation at 14,000 rpm. Cytoplasmic extracts were collected from the supernatant. The pellets were resuspended in buffer B (20 mM HEPES [pH 7.9], 0.42 M KCl, 0.5 mM DTT, 0.2 mM EDTA, and 25% glycerol) and rocked for 20 min 4°C. Nuclear lysates were obtained from the supernatant after centrifugation at 14,000 rpm. An equal amount of cytosolic and nuclear protein was subjected to SDS-PAGE. Membranes were immunoblotted with anti-HuR Ab and anti-TATA box-binding protein (TBP) Ab (to confirm exclusion of nuclear protein from cytoplasmic samples). Membranes were stripped after each Ab treatment with Restore stripping buffer (Thermo Scientific).

### Mice and splenic T cell isolation

The MKK3^−/−^ mice on a C57BL/6 (B6, H-2^b^) background were a generous gift from Dr. Richard Flavell (Yale University, New Haven, CT). The MKK3^−/−^ mice were viable and fertile, with no developmental deficiencies and had normal numbers of thymocytes and splenocytes, and have been described before [39]. C57BL/6 mice were purchased from the Jackson Laboratory (Bar Harbor, ME). Splenic T cells were isolated by negative selection from wt, MKK3^+/−^, or MKK3^−/−^ mice, as described [40]. T cell purity was assessed by Flow cytometry (CD3 positive >95%). All animal experiments were performed in accordance with protocols approved by the Yale University's ethics institutional review board (Institute of Animal care and Use Committee (IACUC); PHS assurance number A3230-10, protocol permit number 2009-10682).

### Immunofluorescence and morphometry

Cells were treated with LFA-1 activation buffer for 60 minutes on rICAM-1 or on PLL coated coverslips. Adhered cells were fixed with 4% paraformaldehyde for 15 minutes at 4°C, and subsequently permeabilized with 0.1% Triton X-100 followed by blocking with 5% goat serum overnight at 4°C. Cells were immunostained with 5 µg/ml anti-HuR or AUF-1 Abs overnight at 4°C followed and co-stained with 0.005% DAPI (4.6 diamidino-2-phenylindole) (Invitrogen, Carlsbad, CA). For the morphometric assay, 25 cells from each condition in 3 different fields were included in the analysis. The ratio of HuR translocation was calculated as the area visible with anti- HuR Abs in the cytoplasm plus the nucleus divided by the nuclear area (by DAPI staining for nuclear definition). The ImageJ software (NIH) was used to determine the areas.

### Statistical analysis

Statistical analysis for the morphometric assay was performed using a Wilcoxon, non-parametric rank-sum test. A p<0.05 was considered significant.

## Supporting Information

Figure S1 HuR translocation in PMA-treated Jurkat cells. A, Jurkat cells were adhered to PLL- or rICAM-1-coated cover slips in the presence or absence of PMA (25ng/ml) for 3h at 37°C, after which they were fixed, permeabilized and immunofluorescent staining was performed with mouse anti-human HuR and anti-mouse Ig-cyanine 5, and with DAPI for nuclear definition. Images were overlaid and merges displayed as noted. Magnification 40X. B, 25 cells in 3 different fields were morphometrically analyzed for HuR nuclear-to-cytosolic translocation and expressed as the ratio of HuR immunofluorescent plus nuclear area divided by the nuclear area only, analyzed by Image J. Each dot represents a single cell ratio. *p<0.05.
(4.20 MB TIF)
